# Call the Psychiatrist! - Study about Delirium in the context of liaison psychiatry

**DOI:** 10.1192/j.eurpsy.2022.613

**Published:** 2022-09-01

**Authors:** I. Fonseca Vaz, S. Mouta, B. Jesus, S. Castro

**Affiliations:** Unidade Local de Saúde da Guarda, Departamento De Psiquiatria E Saúde Mental, Guarda, Portugal

**Keywords:** delirium, symptoms, liaison psychiatry

## Abstract

**Introduction:**

Delirium is characterized as a short-term consciousness and cognition disturbance which tends to fluctuate during the course of the day. It is a common and serious problem, mainly in hospitalized older adults, potentially avoidable and often poorly recognized.

**Objectives:**

We propose an analysis on the theme through a work that evaluates the requests for psychiatric consultation made in a district hospital in Portugal during the course of 12 months.

**Methods:**

We identified all patients on the requests for psychiatric consultation and obtained a demographic, clinical and consultation requests by medical specialties data and conducted statistical analysis using Excel.

**Results:**

We identified 106 consultation requests, in which 41 cases were eventually diagnosed as delirium. Most (83%) were hyperactive delirium, 12% were hypoactive delirium and 5% were mixed delirium. Incidence was higher in males (59%) and in those aged between 66 and 80 years old (56.1%). Most consultation requests were made by Internal Medicine (46.3%), followed by General Surgery (26.8%), Pulmonology (14.6%), Orthopedics (9.8%) and Neurology (2.5%). Finally, we analyzed which symptoms mentioned in the request made physicians consider requesting a psychiatric evaluation. Approximately half of the cases (48.8%) reported psychomotor agitation, followed by temporal/spatial disorientation (41.5%) and aggressive behaviour (17.1%).

**Conclusions:**

We highlight a still notorious lack of proper identification of delirium, resulting in symptoms being incorrectly interpreted as a psychiatric disorder. This may cause a delay in the adequate diagnosis and management of the condition, increasing the morbidity and mortality of patients.

**Disclosure:**

No significant relationships.

